# Clinical Presentation and Tumour Burden in Head and Neck Sarcomas: Implications for Early Diagnosis and Referral

**DOI:** 10.3390/cancers18081298

**Published:** 2026-04-20

**Authors:** Samhita Shanmugasundaram, Malla Salli, Amrita Jay, Antonia Timmis, Xin Kowa, Simon Morley, Katrina Ingley, Rachael Windsor, Ajla Wasti, Harini Rao, Franel Le Grange, Sandra J. Strauss, Vasilios Karavasilis, George Bitar, Simon Wan, Jonathan Joseph, Nicholas Kalavrezos, Deepti Sinha

**Affiliations:** 1 Medical School, University College London, London WC1E 6BT, UK; 2 Department of Head and Neck Surgery, University College London Hospital, London NW1 2BU, UK; 3 Department of Histopathology, University College London Hospital, London NW1 2BU, UK; 4 Department of Radiology, University College London Hospital, London NW1 2BU, UK; 5 London Sarcoma Service, University College London Hospital, London NW1 2BU, UK; 6 Department of Medical Oncology, University College London Hospital, London NW1 2BU, UK; 7 Department of Paediatric Oncology, Great Ormond Street Hospital, London WC1N 3JH, UK; 8 Department of Nuclear Medicine, University College London Hospital, London NW1 2BU, UK; 9 Royal National Ear, Nose & Throat Hospital, London WC1X 8DA, UK

**Keywords:** head and neck sarcoma, diagnostic delay, symptom duration, tumour size, soft tissue sarcoma, bone sarcoma, referral guidelines, early diagnosis, retrospective cohort study

## Abstract

*Background/Objectives:* Head and neck sarcomas account for 11% of all soft tissue and 9% of all bone sarcomas in the UK. Diagnostic delays are common, with non-specific symptoms and histological misdiagnosis reported in up to 42% of cases. This study aims to evaluate the association between presenting symptoms, symptom duration, and tumour size to inform a tailored HNS diagnostic strategy for early referral to a tertiary centre. *Methods:* We analysed a retrospective cohort of 425 adult and paediatric patients referred to the London Sarcoma Service between 2002 and 2025. *Results:* Our cohort analysis identified a median tumour size of 44.00 mm and symptom duration of 3 months. Although symptom duration did not predict tumour size (β = 0.63, *p* = 0.76), non-specific symptoms (swelling, pain, nasal/oral changes) were significantly associated with larger tumours (OR 1.96–3.66), alongside systemic symptoms (β = 22.90 mm, *p* = 0.044). Each 1 mm increase in tumour size was also associated with a 2.60% increased chance of a higher-grade sarcoma (OR = 1.03 per mm, *p* < 0.001). *Conclusions:* To our knowledge, this is the largest cohort study to characterise diagnostic patterns in HNS. Our findings reveal three critical insights: 1. Current size-based referral thresholds are inadequate. 2. Non-specific symptoms, such as nasal or oral symptoms, are frequently overlooked. 3. The anatomical complexity of the HN region demands early tailored diagnostic strategies. We propose a hypothesis-generating ‘1–2–1’ framework to support earlier clinical suspicion, which requires prospective validation.

## Simple Summary

Head and neck sarcomas account for 11% of all soft tissue and 9% of all bone sarcomas in the UK. Head and neck sarcomas (HNS) frequently present as large, locally advanced tumours, requiring complex resections with a high risk of microscopically positive margins (R1) and poor outcomes. However, current UK guidelines do not offer region-specific advice for early detection. This cohort study of 425 patients at a tertiary sarcoma centre aimed to explore the relationship between tumour size, symptoms and symptom duration and assess whether these factors could inform referral criteria for diagnosis. The results revealed that specific symptoms, particularly oral and nasal presentations, were significantly associated with larger tumour sizes, whilst symptom duration showed no consistent correlation. These findings suggest that unexplained symptoms alongside the presence of a mass or swelling in the head and neck region should prompt early radiological evaluation in the primary care setting, with an ultrasound or cone beam CT. These results underscore the need for further refinement of site-specific guidance for this anatomically complex and functionally critical region to prevent diagnostic delays.

## 1. Introduction

### 1.1. Epidemiology and Clinical Burden of Head and Neck Sarcomas

**Figure 1:**
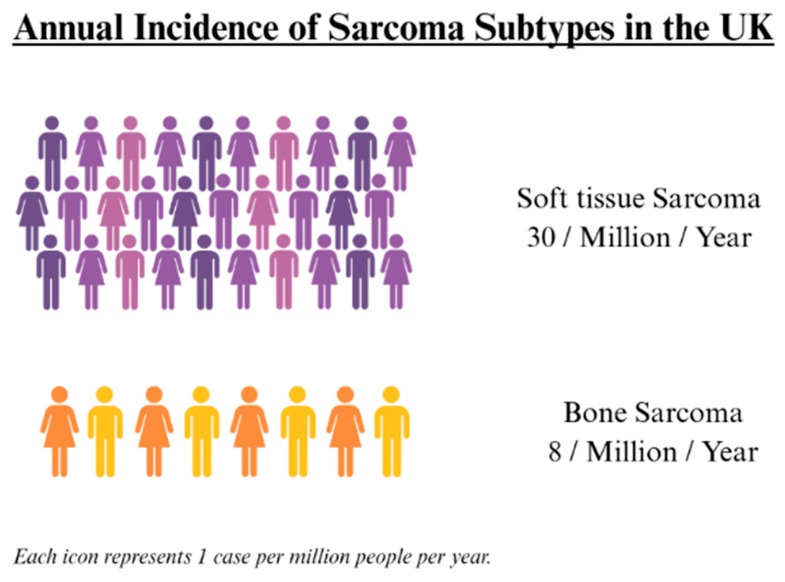
Annual incidence of soft tissue and bone sarcomas in the UK. Soft tissue sarcomas occur at approximately 30 cases per million per year, and bone sarcomas at 8 per million. Each icon represents one case. Data source: Sarcoma UK. Created in BioRender.

Head and neck sarcomas (HNS) are rare, heterogeneous mesenchymal malignancies, comprising 11% of all soft tissue sarcomas (STS) and 9% of all bone sarcomas (BS) [1]. Diagnostic delays are common, with non-specific symptoms and histological misdiagnosis reported in up to 42% of cases [2,3]. The annual incidence of STS and BS is approximately 30 and 8 cases per million, respectively [1,4,5,6] (Figure 1).

Due to their histological complexity and need for multimodal treatment, the National Health Service (NHS) specification mandates that all patients with suspected sarcomas are referred to the 15 centralised specialist multidisciplinary teams (MDTs) to ensure equitable access to expert services, though diagnostic delays persist across sites [5,7,8,9].

### 1.2. Diagnostic Challenges

Patients with HNS present with heterogeneous and nonspecific symptoms, contributing to diagnostic delays [10,11]. While 80% of STS present as painless lumps, osteosarcomas often present with pain [12]. However, this distinction is not absolute. Studies report under-recognised symptoms, such as unilateral sinusitis, otalgia, cranial nerve deficits, and tooth mobility (13), alongside signs such as voice changes and dysphagia [5] that warrant urgent referral but are frequently missed. Despite their clinical relevance, current guidelines do not highlight these symptoms, contributing to delays.

Compounding this challenge, the region’s anatomical complexity means even small tumours have a significant functional and aesthetic impact [13]. Complete surgical excision with negative margins (R0) is required for a cure, necessitating extensive resection, complex reconstruction, and multimodal therapy [12]. Consequently, prognosis remains poor [14].

**Figure 2:**
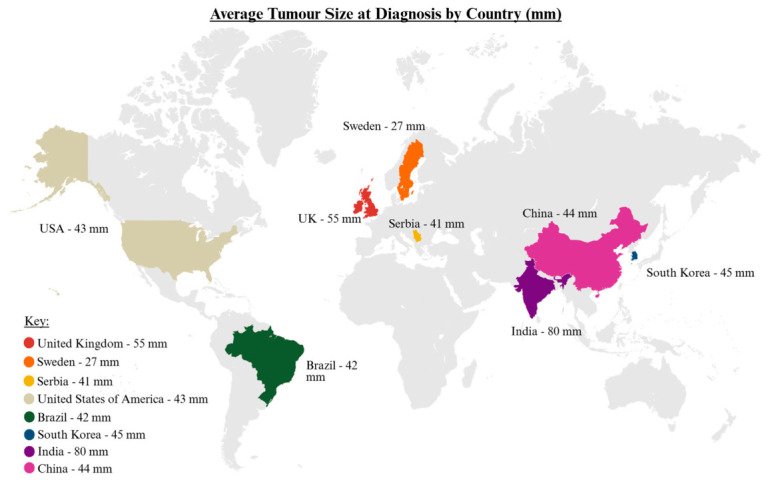
Mean tumour size at diagnosis by country for head and neck sarcomas, based on published cohort data [16,17,18,19,20,21,22,23,24,25,26,28,29,30]. Created in BioRender.

In the UK, the mean tumour dimension at initial diagnosis is 55mm, presenting as locally advanced disease. In contrast, outside the UK, the average tumour dimensions are 27 mm (Sweden), 42 mm (Brazil), 44 mm (China), and 45 mm (South Korea) at diagnosis [15,16,17,18,19,20,21,22,23,24,25,26,27,28,29,30] (Figure 2). This suggests that community awareness and current referral protocols in the UK may be inadequate and require revision.

### 1.3. Limitations of Referral Guidelines

UK sarcoma referral guidelines historically emphasise tumour size, pain, and growth as referral criteria.

The 2006 National Institute for Health and Care Excellence (NICE) CSG9 criteria recommended urgent referral for soft tissue masses that are painful, greater than 5 cm, increasing in size, or deep to the fascia [31,32]. This is reaffirmed by the British Sarcoma Group (BSG) in 2016 and 2019, primarily for limb and trunk sarcomas [33,34]. The 2015 NICE NG12 revision and 2024 BSG consensus both emphasised “unexplained lump increasing in size,” signalling a shift toward symptom progression-based referrals rather than static thresholds [8,35]. While they advise consulting site-specific guidance for soft-tissue sarcomas, none of these documents provides such guidance [8,33].

**Figure 3:**
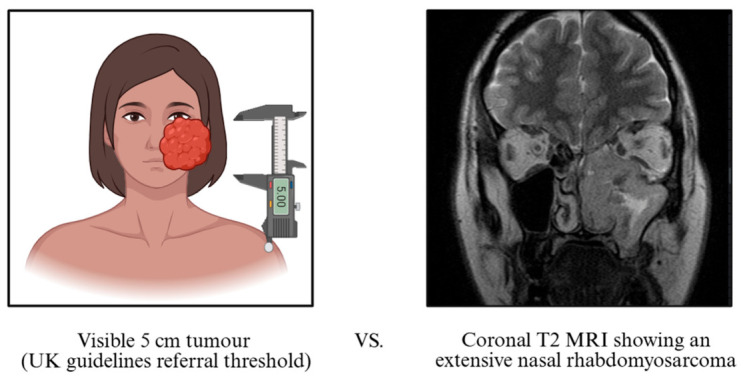
Illustration of limitations of size-based referral criteria. (**Left**): Schematic representation of a visible 5 cm facial tumour. (**Right**): Coronal T2-weighted MRI showing an extensive midface sarcoma without an obvious external mass. Image adapted from Radiopaedia (case ID 183462) [36]. NICE CSG9 and BSG 2019 referral criteria [34,35]. Created in BioRender.

The >5 cm threshold may be appropriate in extremities, but is poorly suited to the anatomically constrained HN region, where smaller lesions can be extensive. Figure 3 exemplifies this diagnostic challenge, illustrating a midface sarcoma with substantial infiltration that was externally inconspicuous and below the size threshold. Moreover, “increasing size” is inconsistently documented in practice, likely due to subjective interpretation or slow tumour growth.

Osteosarcoma guidelines highlight pain, a lump, or swelling, especially night-time pain, as red flags, but offer no tailored symptomology accounting for the complex HN anatomy [37,38].

Diagnostic delays in HNS remain a significant challenge, driven by nonspecific symptoms, a lack of region-specific referral guidance, and inconsistent recognition of clinical red flags, which lead to late referrals and larger tumours. Compounding this, a paucity of evidence linking symptom patterns, duration, and tumour progression hinders the development of targeted early detection tools. Together, these underscore the urgent need for evidence-based, site-specific referral criteria to improve diagnostic timelines, standardise care, and enhance patient outcomes in the UK.

This study aims to define the diagnostic presentation of head and neck sarcomas and assess the adequacy of current size-based referral criteria in this anatomically complex region.

Specifically, we aim to:

1. Identify and categorise commonly reported symptoms of HNS in the UK;
2. Examine the relationship between symptom type, duration, and tumour size at diagnosis;
3. Explore whether specific symptom patterns or tumour size distributions can inform early diagnostic decision-making and support the development of HNS referral criteria.

## 2. Materials and Methods

### 2.1. Data Source and Study Population

A retrospective cohort analysis was conducted using anonymised records from patients presenting to the London Sarcoma Service (LSS) between 2002 and 2025. The study period was limited to 2002 onwards to reflect the availability of consistent electronic patient records and contemporary diagnostic pathways. All patients gave consent to NHS data sharing, and all radiological images published are anonymised. This cohort included 425 patients of all ages diagnosed with HNS, confirmed with dual histopathological analysis and next-generation sequencing (NGS) where available.

### 2.2. Data Collection and Extraction

Data were extracted from EPIC electronic health records using a standardised template. Variables included:

- Demographic variables: Age at diagnosis, sex, and survival status;
- Tumour characteristics: Histological subtype, anatomical site, maximum tumour diameter in any plane (extracted from Magnetic Resonance imaging [MRI], radiology reports, dual pathology reports, and next-generation sequencing [NGS] reports), and tumour grade (Fédération Nationale des Centres de Lutte Contre le Cancer system, FNCLCC);
- Clinical presentation: Presenting symptoms and symptom duration.

Symptom categories were defined in advance based on clinically relevant groupings (e.g., nasal, oral, neurological) to reflect real-world presenting complaints (Table 1). Tumour sites were also grouped into predefined anatomical categories based on surgical zones to standardise heterogeneous anatomical descriptions across radiological and operative reports (Table 2).

Maximum tumour diameter was selected over volume or visible size, due to its consistent availability and to reduce confounding from multicompartment involvement. The proportion of tumours measuring <5 cm was calculated using the recorded maximum tumour diameter at diagnosis. Tumour size analyses were performed on patients with available maximum tumour diameter data, and cases with missing tumour size were excluded from size-based analyses.

### 2.3. Statistical Analysis

Descriptive statistics, including means, standard deviations (SD), medians, interquartile ranges (IQR), frequencies, and percentages, were calculated using R (version 4.4.1; R Foundation for Statistical Computing, Vienna, Austria) with supplementary data handling performed in Microsoft Excel.

Group comparisons were performed using the Mann–Whitney U test for continuous variables with non-normal distribution (e.g., tumour size and symptom duration between adult and paediatric patients), and chi-squared tests for categorical variables (e.g., sex, grade, tumour site, and histological subtype).

Subgroup analyses assessed tumour size and symptom duration across histological subtypes and grades using visual summaries and Kruskal–Wallis testing. Descriptive comparisons examined the relationship between tumour grade and tumour size categories (e.g., <30 mm vs. >60 mm). Ordinal logistic regression evaluated the association between continuous tumour size and ordinal tumour grade (1–3), reporting odds ratios (OR) per mm increase.

#### 2.3.1. Regression Analyses

Multivariable regression analysis was performed as an exploratory, hypothesis-generating approach to assess associations between presenting symptoms and tumour size. Variables were selected based on clinical relevance.

Both multivariable logistic regression (tumour size > 50 mm) and linear regression (continuous tumour size) were performed. Each symptom was entered as a binary covariate. Symptom duration was log-transformed to reduce skew and included as a predictor in both models.

Formal assessment of multicollinearity and application of variable selection methods were not performed.

#### 2.3.2. Statistical Considerations

Missing data were handled using complete case analysis. No correction for multiple testing was applied, given the exploratory nature of the analysis and limited power in this rare disease cohort. All tests were two-tailed with significance set at *p* < 0.05. Regression results were visualised using forest plots where appropriate. Key limitations of this dataset include the inability to determine a time-to-death or assess its impact on outcomes due to missing national registry linkage and potential confounding from the COVID-19 pandemic.

## 3. Results

### 3.1. Patient and Tumour Characteristics

A total of 425 patients diagnosed with HNS were identified from the LSS treatment database. Of these, 234 were male (55.1%) and 191 were female (44.9%). The mean age at diagnosis was 46.7 years, 80.2% ≥24 years, and 19.8% <24 years. Patients and tumour characteristics are summarised in Table 3. Tumour classifications include STS (54.6%), BS (36.2%), and dermal sarcomas (DS) (9.2%). Grade data were available for 318/425 cases, with missing data attributed to absent reporting or ungradable subtypes. Grade 3 tumours predominated (49.9%), followed by Grade 1 (16.7%) and Grade 2 (10.4%).

#### 3.1.1. Histological Subtype

**Figure 4:**
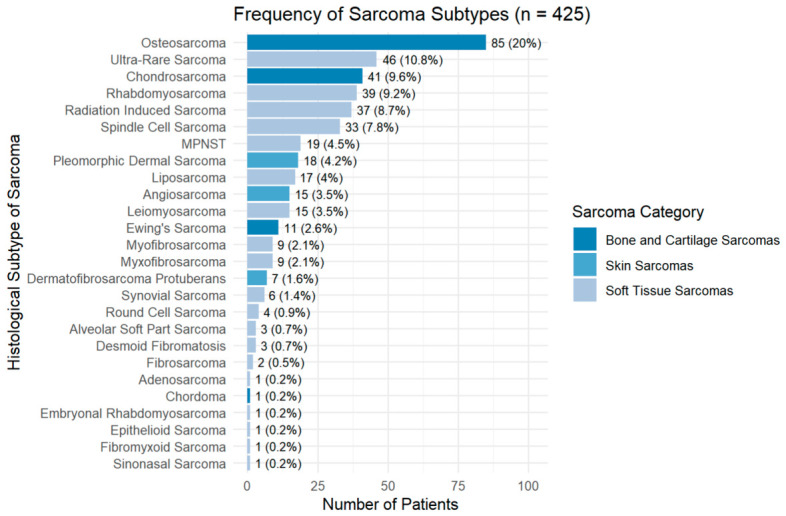
Distribution of histological subtypes in the cohort (*n* = 425). Subtypes with fewer than five cases are shown for completeness (<1% each).

Osteosarcoma was the most frequent subtype (20%), followed by ultra-rare soft-tissue phenotypes (10.8%) and chondrosarcoma (9.6%) (Figure 4).

#### 3.1.2. Tumour Site

**Figure 5:**
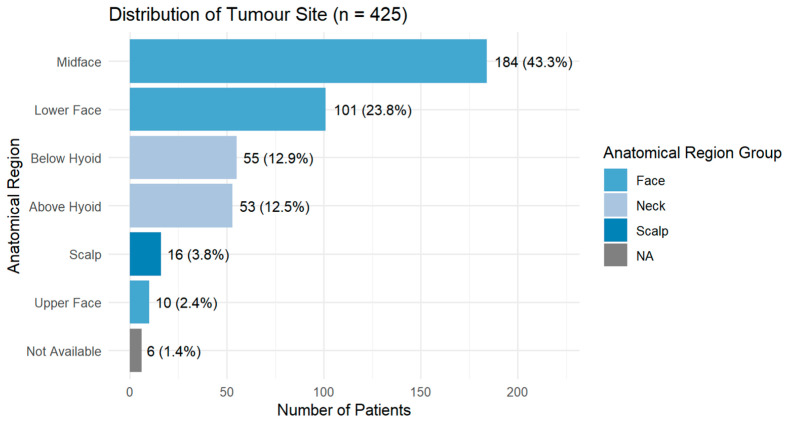
Distribution of tumour anatomical sites in the cohort (*n* = 425). In six cases (1.4%), the anatomical site could not be determined due to incomplete records.

The majority of tumours were in the midface (43.3%), followed by the lower face (23.81%) and neck below the hyoid (12.9%). Upper-face tumours were the least frequent (2.4%) (Figure 5).

### 3.2. Tumour Size

Tumour size was recorded for 340 patients. Among these, 52.1% (*n* = 177) had tumours measuring <5 cm, while 47.9% (*n* = 163) measured ≥5 cm at diagnosis. The mean tumour size was 48.9 mm (SD = 27.3), with a median of 44 mm (IQR: 30–65 mm). There was no significant difference in tumour size between adult and paediatric populations (W = 2966.5, *p* = 0.448) (Table 4).

#### 3.2.1. Tumour Size Across Subtype

**Figure 6:**
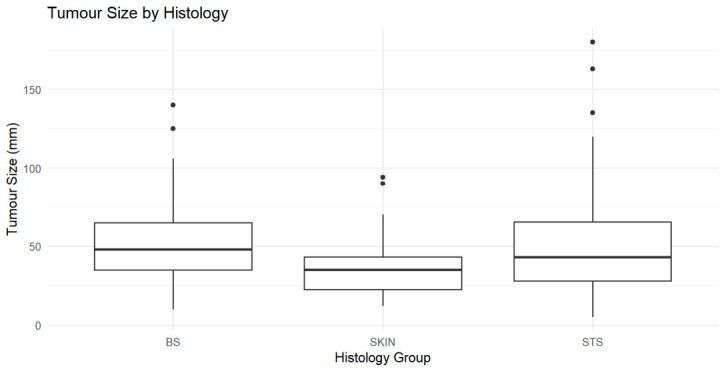
Tumour size distribution across major sarcoma categories (bone, dermal, and soft tissue). Boxplots show median and interquartile range; points represent outliers.

**Figure 7:**
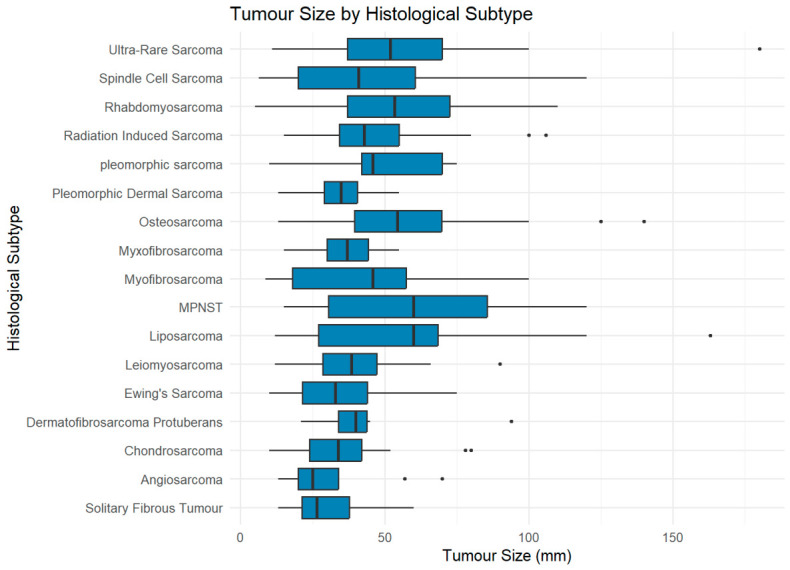
Tumour size distribution by histological subtype. Boxplots show median and interquartile range; points represent outliers beyond 1.5× IQR.

Tumour size varied across histological groups, with soft tissue sarcomas generally presenting with larger tumours. Subtype-level analysis demonstrated heterogeneity, with MPNST, liposarcoma, and osteosarcoma showing wider size distributions and higher medians, while subtypes such as pleomorphic sarcoma and dermatofibrosarcoma protuberans exhibited more tightly clustered sizes (Figure 6 and Figure 7).

#### 3.2.2. Tumour Size Across Grade

**Figure 8:**
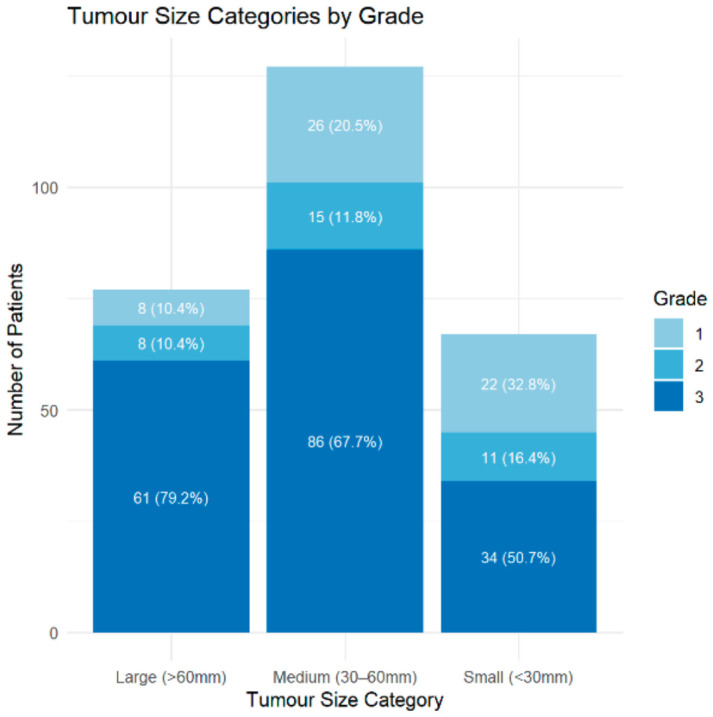
Distribution of tumour grade across size categories (<30 mm, 30–60 mm, >60 mm). Percentages shown within bars represent the proportion of each grade within the size category.

Larger tumours (>60 mm) were predominantly Grade 3 (79.2%), while smaller tumours (<30 mm) were more often Grade 1 (32.8%) (Figure 8).

**Figure 9:**
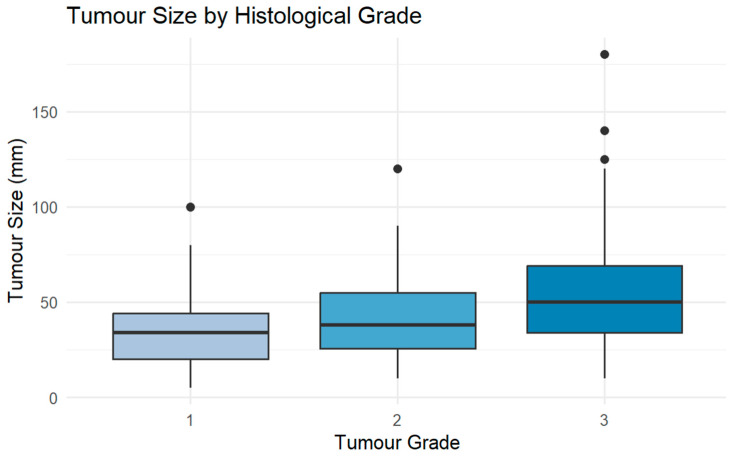
Tumour size distribution by histological grade. Boxplots show median and interquartile range; points represent outliers.

Figure 9 illustrates the distribution of tumour size across histological grades, with median tumour size increasing progressively with grade. Ordinal logistic regression confirmed a statistically significant association: each 1 mm increase in tumour size was associated with a 2.6% increase in the odds of a higher-grade tumour (OR = 1.03 per mm, 95% CI: 1.01–1.04, *p* < 0.001).

### 3.3. Symptoms

**Figure 10:**
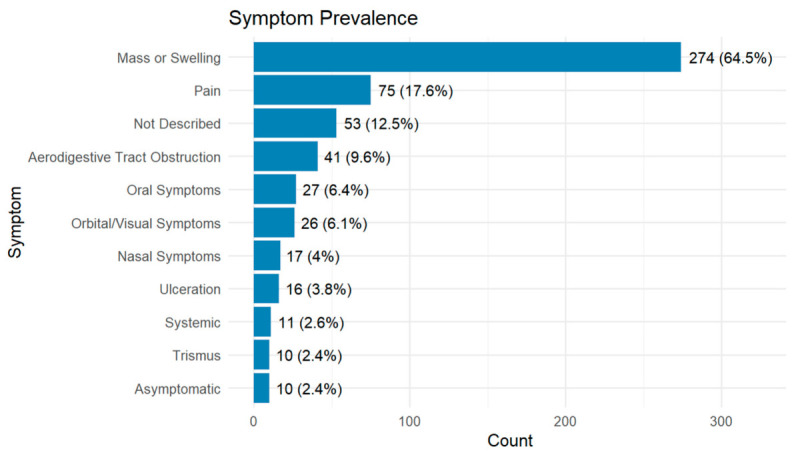
Frequency of presenting symptoms in the cohort (*n* = 425).

Mass or swelling was the most frequently reported symptom (64.5%), followed by pain (17.6%) and aerodigestive tract obstruction (9.6%). Less common symptoms included oral symptoms (6.4%), orbital or visual disturbances (6.1%), and nasal symptoms (4.0%). Ulceration, trismus, and systemic symptoms each occurred in <5% of cases. Only 2.4% of patients were asymptomatic, and symptom data were missing in 12.5% of cases (Figure 10, Appendix A, Table A1).

#### Symptoms Prevalence by Histological Subtype

**Figure 11:**
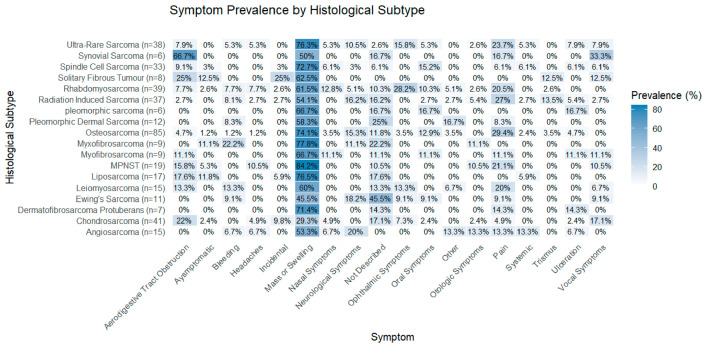
Heatmap showing prevalence of presenting symptoms across histological subtypes. Darker shading indicates higher symptom prevalence.

Symptom profiles varied across histological subtypes (Figure 11). Ultra-rare sarcomas most frequently presented with mass or swelling (76.3%), while synovial sarcoma showed high rates of aerodigestive tract obstruction (66.7%) and vocal symptoms (33.3%). Rhabdomyosarcomas and Ewing’s exhibited broader symptom profiles, whereas subtypes like Dermatofibrosarcoma Protuberans showed narrower symptom profiles.

### 3.4. Symptom Duration

Symptom duration varied widely (mean = 11.4 months, SD = 37.4), with a median of 3 months (IQR: 1–6 months), reflecting a right-skewed distribution due to a few prolonged cases (up to 481 months) (Table 5). A Mann–Whitney U test showed significantly longer durations in adults compared to paediatric patients (W = 5178.5, *p* = 0.006), indicating potential diagnostic delays.

**Figure 12:**
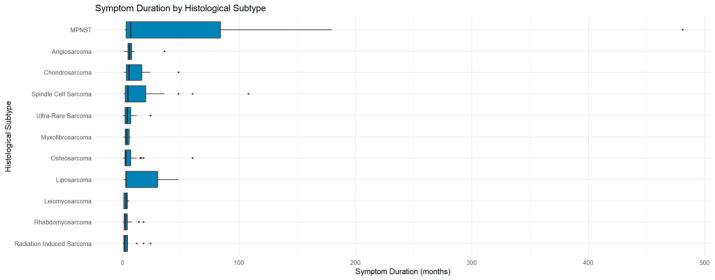
Distribution of symptom duration across histological subtypes. Boxplots show median and interquartile range; points represent outliers. Only subtypes with sufficient data are included.

Duration also varied significantly across histological subtypes (Figure 12; Appendix A, Table A2), ranging from 2 months in radiation-induced sarcomas to 7 months in MPNSTs. A Kruskal–Wallis test confirmed this variability (χ^2^ = 23.82, df = 10, *p* = 0.008), reflecting differences in tumour behaviour or diagnostic pathways.

### 3.5. Predictors of Larger Tumour Size

To identify predictors of large tumour size (>5 cm), a multivariable logistic regression was performed. Mass or swelling (OR = 2.95, 95% CI: 1.78–5.02, *p* < 0.001), pain (OR = 1.96, 95% CI: 1.15–3.35, *p* = 0.014), oral symptoms (OR = 2.81, 95% CI: 1.24–6.50, *p* = 0.013) and nasal symptoms (OR = 3.66, 95% CI: 1.24–11.0, *p* = 0.019) were significantly associated with increased odds. Symptoms such as ulceration, systemic symptoms, or visual symptoms were not significantly associated (*p* > 0.05) (Table 6).

A parallel multivariable linear regression showed that systemic symptoms were significantly associated with larger tumours (β = 22.90 mm, *p* = 0.044), while mass or swelling (β = 8.33 mm, *p* = 0.096) and nasal symptoms (β = 14.58 mm, *p* = 0.119) showed non-significant positive trends (Table 7). The model explained a small proportion of the variance in tumour size (adjusted R^2^ = 0.027) and was not statistically significant overall (F(8, 187) = 1.67, *p* = 0.109).

### 3.6. Association Between Symptom Duration and Tumour Size

**Figure 13:**
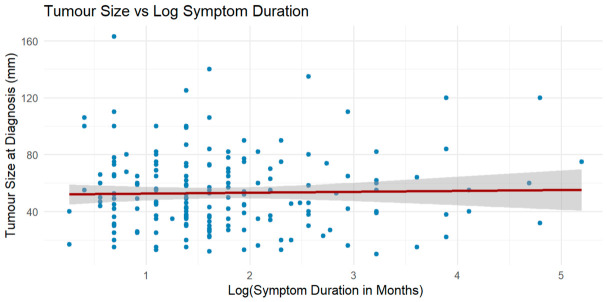
Relationship between tumour size and log-transformed symptom duration. Each blue dot represents an individual patient. The red regression line and shaded 95% confidence interval represent the linear model fit.

Linear and logistic regression found no significant association between log-transformed symptom duration and tumour size. Duration was not predictive of tumour size (β = 0.63, *p* = 0.758, R^2^ < 0.001) nor of having a tumour >5 cm (OR = 1.12, *p* = 0.454) (Table 8, Figure 13). These findings suggest symptom duration alone is not a reliable predictor of tumour size in this cohort.

## 4. Discussion

Head and neck sarcomas are a rare entity that frequently presents with vague symptoms. The eighth edition of the TNM represents an important step forward by classifying head and neck bone sarcomas within the axial-skeletal system, and for the first time, recognising head and neck soft tissue sarcomas as an independent entity, albeit with little guidance [39]. Moreover, extrapolating data from other common sites may not provide early diagnosis or improve treatment in rare diseases.

In this context, the present large retrospective study provides a unique opportunity to investigate the current gap in knowledge in early diagnosis. To our knowledge, this is the first study to systematically characterise diagnostic presentation in head and neck sarcomas, including the relationship between presenting symptoms, symptom duration, and tumour size, while critically evaluating the limitations of current size-based referral criteria, variability in first-line imaging, and the absence of tailored guidance for primary care clinicians.

Our findings demonstrate that current diagnostic pathways are associated with an unacceptably low and late sarcoma detection rate. This contributes to both delayed presentation with larger tumours and advanced disease, as well as the inefficient use of specialist resources through inappropriate referrals of non-sarcoma pathology. Together, these factors perpetuate a resource-intensive system that lacks effective triage at the point of initial clinical contact.

These findings highlight limitations in current diagnostic and referral approaches, which may not adequately reflect the unique clinical behaviour of head and neck sarcomas. Drawing on our cohort analysis, we present a pragmatic, symptom-led conceptual framework designed for use in primary care settings. This framework should be interpreted as exploratory and hypothesis-generating, and requires prospective validation prior to clinical implementation.

### 4.1. Limitations of Current Referral Guidelines

A key issue is the absence of UK referral guidelines for HNS [8,33,37]. Our findings challenge reliance on tumour size and pain–referral criteria endorsed by NICE [31], which fail to capture the clinical realities of HNS. Over a third of patients in our cohort had tumours < 5 cm with significant functional/aesthetic impairment.

We identified a broader set of symptoms meaningfully associated with tumour size or clinical severity. Oral/nasal symptoms, systemic symptoms, neurological symptoms, and aerodigestive tract obstruction were often observed in tumours < 5 cm, underscoring the limitations of rigid size-based thresholds in the HN region.

Symptom patterns varied by histological subtype: STS caused more functional deficits, while BS often presented with swelling or pain. Pain was more frequent in sarcomas affecting innervated bones (e.g., trigeminal innervation of the mandible), potentially explaining the recognition of night pain as a red flag in osteosarcomas [37,38].

These results suggest that symptom diversity and anatomical context, not size or pain alone, should guide referrals.

### 4.2. Limitations of Imaging

Current clinical practice is plagued by inappropriate imaging and interventions. Whilst international guidelines recommend ultrasound, CT, and MRI for initial assessment, adherence is variable [40,41].

**Figure 14:**
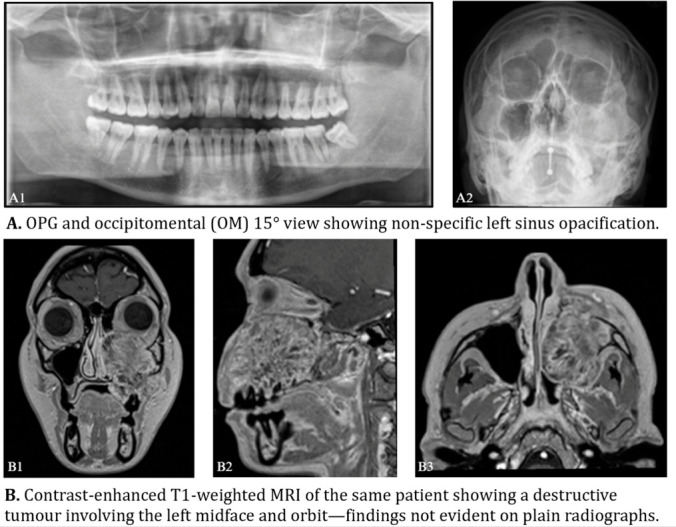
Comparison of plain radiography and cross-sectional imaging in a head and neck sarcoma. (**A1**,**A2**) Orthopantomogram and occipitomental radiographs showing left maxillary sinus opacification. (**B1**–**B3**) Contrast-enhanced T1-weighted MRI demonstrating a locally invasive soft tissue mass involving the left midface with extension into adjacent structures. Images reproduced with permission from NHS archives.

Diagnostic delays are frequently driven by the underuse and misinterpretation of imaging in primary care. Clinically, primary imaging in suspected HNS often relies on plain skull X-rays or orthopantomograms [OPGs], despite their low sensitivity for early-stage tumours. These can miss key features such as bone destruction and soft tissue swelling, making confident inclusion or exclusion of suspected sarcoma challenging and increasing the need for subsequent sensitive imaging (Figure 14) [14]. Two-dimensional radiographs (e.g., skull X-rays or OPGs), whilst acceptable for the mandible, are inadequate for midface evaluation, as they fail to detect early bony destruction or soft tissue invasion (e.g., sunburst periosteal reactions in osteosarcoma).

Consistent with this, our cohort revealed frequent reliance on two-dimensional (2D) imaging for jaw or oral cavity symptoms, despite their inability to visualise three-dimensional (3D) anatomy or early tumour invasion [42]. For example, dental practices often rely on 2D dental X-rays, which are inadequate for early sarcoma detection [42]. Although CBCT offers high spatial resolution, a short scan time, and is widely available in primary dental care settings, it remains underused and frequently misinterpreted, leading to misdiagnoses (e.g., as dental abscesses) and inappropriate interventions like tooth extractions, which may exacerbate tumour growth [43].

Inadequate initial imaging frequently necessitates repeat scans and delayed referrals. We therefore advocate for revised imaging protocols that prioritise high-resolution modalities in cases of persistent or unexplained HN symptoms, and targeted training to improve early identification of high-risk radiological features and better assessment of local invasion.

Further, inappropriate early interventions, such as excisions performed without prior imaging, diagnosis, or biopsy, were also common and often driven by entrenched benign-lumpectomy practices in non-specialist surgical settings. Similarly, there was a clinical overuse of fine needle aspiration cytology (FNAC), despite its limited diagnostic utility in sarcoma, and evidence demonstrating significantly lower diagnostic accuracy and histological concordance compared with core needle biopsy [44]. Patients frequently presented to specialist services with advanced disease following cosmetic or partial excisions (“whoops” procedures), highlighting the downstream consequences of inappropriate diagnostic and biopsy practices. In a published cohort study, 42% of patients required re-excision after referral to a head and neck sarcoma MDT, with 13% subsequently requiring adjuvant chemotherapy and 17% postoperative radiotherapy, underscoring the morbidity associated with non-specialist intervention [45].

Collectively, these findings emphasise the critical need for adequate cross-sectional imaging, image-guided core biopsy, and early referral to specialist MDTs in suspected head and neck sarcomas. These recommendations reflect expert-informed guidance and are not directly derived from comparative analysis within this cohort.

### 4.3. Persistent Non-Specific Symptoms and Diagnostic Challenges

A proportion of HNS patients present with persistent, non-specific symptoms that are often overlooked in primary care. These include unilateral sinusitis and progressive midface swellings, often exhibited by orbital, nasal, or aerodigestive tract obstruction symptoms, which may initially appear benign but often represent early signs of aggressive pathology and may later be identified at an unresectable stage (>5 cm).

Crucially, unilateral symptoms and symptoms in the midface, such as rhinorrhoea, nasal obstruction, or facial asymmetry, should prompt urgent cross-sectional imaging (CT/MRI) to exclude malignancy. By the time patients develop advanced signs like orbital proptosis or palpable masses (>5 cm), tumours are frequently unresectable.

This clinical progression, from initial subtle symptoms to advanced disease, highlights several important practice implications: primary care providers should maintain a low threshold for requesting imaging in cases of persistent (>2 months duration) unilateral symptoms, as the window for detecting resectable disease may be narrow. Timely referral is crucial, and education campaigns should emphasise these early warning signs among frontline clinicians.

### 4.4. Symptom Duration

Prolonged symptom duration remains a hallmark of diagnostic delay in HNS. The median symptom duration in our cohort was 3 months (mean: 11.4 months). Adults experienced longer delays with longer reported symptom duration to diagnosis (median 4.0 months, IQR 2.0–8.5; mean 13.2 months) than children (median 3.0 months, IQR 1.3–4.5; mean 4.6 months). Despite shorter symptom durations, paediatric patients often presented with larger tumours, likely reflecting more aggressive histopathological subtypes. A compounding factor could be a lack of accurate patient histories. Misdiagnosis as benign, incomplete excision, and subsequent radiotherapy were common in children, raising concerns about long-term risks such as radiation-induced sarcomas.

Importantly, symptom duration was not a reliable predictor of tumour size, and duration also varied significantly by histological subtype in our results. As a clinical variable, it is subject to recall bias, inconsistent documentation, and variable attribution and should therefore be interpreted as a broad clinical indicator rather than a precise predictor. These findings suggest that relying on symptom duration as a proxy for disease severity may be misleading, particularly for tumours with rapid growth or deep anatomical locations, where seemingly innocuous symptoms (e.g., nasal congestion or dysphonia) may be overlooked.

### 4.5. Grading and Histological Classification

Due to the 2018 TNM reclassification and the use of multiple grading systems (e.g., FNCLCC, TNM) across studies, an analysis of tumour grade could not be performed [46,47]. However, our cohort showed that larger tumours were significantly more likely to be high-grade (Grade 3), aligning with their known metastatic potential and poorer outcomes [48,49]. The mean tumour size in our cohort (48.9 mm ± 27.3 mm) exceeded international benchmarks [16,17,18,19,20,21,22,23,24,25,26,28,29,30], suggesting a lack of systemic recognition and diagnostic delays.

### 4.6. A Conceptual Framework for Early Referral—The 1–2–1 Rule

Drawing on these findings, we present a conceptual ‘1–2–1’ framework (≥1 symptom, ≥2 months duration, with or without ≥1 cm mass) to support earlier clinical suspicion in head and neck sarcomas. This framework is hypothesis-generating and has not been formally validated, and should be interpreted as a prompt for further evaluation rather than a diagnostic tool. The proposed thresholds are clinically informed and supported by observed associations within the cohort, but were not derived from formal diagnostic modelling.

**Figure 15:**
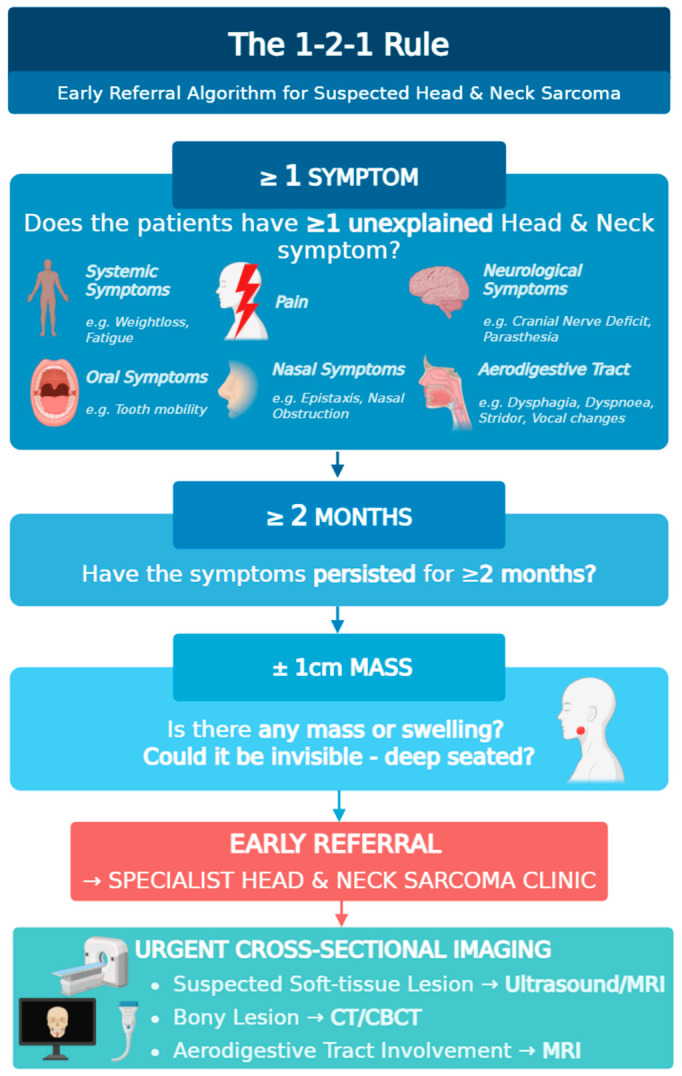
The 1–2–1 conceptual framework for early referral in suspected head and neck sarcomas. The framework incorporates symptom presence, duration, and associated mass. Created in BioRender. Shanmugasundaram, S. (2026).

Figure 15 provides a visual overview of the 1–2–1 framework, defined by the following criteria:

1. Presence of at least one persistent non-specific symptom (pain, aerodigestive tract obstruction, oral, nasal, systemic and/or neurological symptoms),
2. Symptom persistence for ≥2 months, and
3. The presence or absence of an associated mass or swelling of ≥1 cm.

Patients meeting these criteria may benefit from referral to a sarcoma specialist centre for further investigation and imaging by designated practitioners. Where imaging is accessible within primary care, potential first-line cross-sectional imaging modalities include ultrasound (US) for suspected soft tissue lesions and CT or CBCT for bony lesions or suspected upper aerodigestive tract involvement [42,50,51]. In cases of diagnostic or radiological uncertainty or where access to imaging is limited, direct referral to a tertiary centre for multidisciplinary team (MDT) review may be appropriate.

Designed for simplicity and ease of adoption, the conceptual 1–2–1 rule reflects real-world symptomatology and may support timely referral without substantial resource burden. Diagnostic performance metrics (e.g., sensitivity, specificity, predictive values) were not evaluated, and prospective validation is required. Implementation of such frameworks must consider potential increases in referral burden and should be applied in conjunction with clinical judgement and appropriate use of early imaging. These findings should inform, rather than directly dictate, referral behaviour.

### 4.7. Strengths and Limitations

This study represents one of the most comprehensive evaluations of diagnostic presentation in UK HN patients, encompassing both soft tissue and bone sarcomas across paediatric and adult populations. The breadth of this cohort enhances the generalisability of these findings within this anatomically and clinically complex region.

As with any retrospective study conducted in a tertiary referral centre, limitations include potential selection bias, as the cohort is likely enriched for more complex or advanced cases, which may limit generalisability to primary care settings. Incomplete external radiology reports, particularly with respect to tumour size, and missing symptom duration data in primary care referrals further limited data completeness. This analysis was exploratory and hypothesis-generating; formal assessment of multicollinearity and the use of variable selection techniques were not performed, which may introduce model instability. The low explanatory power (adjusted R^2^ = 0.027) likely reflects the multifactorial determinants of tumour size in head and neck sarcomas, influenced by tumour biology, anatomical constraints, and healthcare access. Residual confounding due to histological heterogeneity is also likely, given the inclusion of diverse sarcoma subtypes. Additionally, temporal changes in imaging, pathology classification, grading systems, and referral pathways over the study period may introduce further heterogeneity and confound observed associations.

These findings highlight the need for prospective, standardised data collection and consistent reporting to improve early detection and guide the development of validated diagnostic tools.

### 4.8. Future Directions

Our findings highlight the need for improvements in HNS diagnosis, particularly for subtypes such as rhabdomyosarcoma, myxofibrosarcoma, MPNST, and leiomyosarcoma, which are consistently diagnosed late. Nationally, HN-specific referral guidelines should be developed using empirical evidence and adapted from frameworks such as NCCN and ESMO, as current guidelines lack specificity [8,38,40,41,52,53].

Our proposed 1–2–1 triage tool requires validation through clinician-patient focus groups and cluster randomised trials against current NICE pathways [35]. Prospective multicenter studies are needed to assess the diagnostic accuracy (sensitivity and specificity), clinical impact on time-to-diagnosis, and the cost-effectiveness, including estimates of the number needed to test (NNT). Implementation should involve standardised first-line imaging protocols (US, CBCT, and MRI) which offer superior spatial resolution for maxillofacial pathology [42,51].

Further, educational modules for general practitioners (GPs) and dentists to increase awareness, rapid-access diagnostic hubs supported by telemedicine, imaging referral criteria, and structured GP referral letters are essential to improve awareness and ensure consistent documentation of symptom onset, duration, and functional impact.

## 5. Conclusions

To our knowledge, this comprehensive cohort analysis is one of the largest to evaluate diagnostic patterns in head and neck sarcomas and the first of its kind to investigate a potential relationship between symptoms, duration, and tumour size.

Our findings reveal three critical insights:

First, current size-based referral thresholds are inadequate. In our cohort, 37% of clinically significant tumours were smaller than 5 cm, and each 1 mm increase in size was associated with a 2.6% increase in the odds of a higher-grade tumour (*p* < 0.01). Symptom duration was not associated with tumour size, indicating that time alone is a poor diagnostic marker.

Second, non-specific symptoms (e.g., pain, nasal or oral) were frequently overlooked, despite their association with larger tumours (OR 1.96–3.66).

Third, the anatomical complexity of the head and neck region demands tailored diagnostic strategies. We propose: (1) prompt imaging (US, CT, or MRI) for persistent nonspecific symptoms lasting >2 months; (2) replacement of size thresholds with symptom-based criteria; and (3) development and dissemination of region-specific referral guidelines with imaging recommendations in educational seminars.

Implementation of these measures could substantially reduce diagnostic delays while optimising resource utilisation for this rare but clinically significant cancer. However, further validation through multicentre prospective studies is essential to establish standardised referral pathways that reflect the distinctive biological behaviour and anatomical complexity of HNS.

## Figures and Tables

**Table 1:** Symptom categories used for data extraction. Presenting symptoms were grouped into 12 predefined categories based on clinical relevance and frequency in the literature, with examples.

Symptom Category	Definition/Examples
Mass or Swelling	Palpable lump, visible swelling
Pain	Localised or referred pain
Ulceration	Mucosal or cutaneous breakdown
Oral Symptoms	Tooth mobility, oral discomfort, swelling, increased interdental spacing
Trismus	Reduced mouth opening
Aerodigestive Obstruction	Dysphagia, dyspnoea, stridor
Nasal Symptoms	Nasal obstruction, discharge
Orbital or Visual Symptoms	Diplopia, proptosis, vision changes
Neurological Symptoms	Cranial nerve palsy, sensory loss
Systemic Symptoms	Weight loss, fever, fatigue
Asymptomatic	No reported symptoms
Not Described	No symptom documentation available

**Table 2:** Anatomical classification of tumour sites based on surgical zones. Used to group tumour location for consistency across radiological and operative reports.

Site Category	Definition/Examples
Upper Face	Frontal bone, forehead, upper orbit, scalp
Mid Face	Nasal cavity, maxilla, zygoma, ethmoid, orbit, paranasal sinuses, skull base, hard/soft palate
Lower Face	Mandible, buccal mucosa, lips, floor of mouth, tongue
Suprahyoid	Tongue base, tonsil, oropharynx, submandibular space, parapharyngeal space
Infrahyoid	Larynx, hypopharynx, cervical soft tissues, thyroid, supraclavicular and posterior triangle of neck
Other	Tumours crossing multiple zones or with an unclear site of origin

**Table 3:** Patient and tumour demographic characteristics from the London Sarcoma Service head and neck sarcoma cohort (*n* = 425), 2002–2025. Tumour grade was unavailable in 25.9% of cases due to incomplete records or limitations in grading applicability.

Patient Demographics	
Total Patients	425
Age (mean ± SD)	46.7 ± 22.6
Age (IQR)	27.8–66.0
Adults (≥24 years)	341 (80.2%)
Paediatric and TYA (<24 years)	84 (19.8%)
Male	234 (55.1%)
Female	191 (44.9%)
Tumour classification	Frequency, *n* (%)
STS	232 (54.6%)
BS	154 (36.2%)
Dermal	39 (9.2%)
Tumour Grade	Frequency, *n* (%)
1	69 (16.7)
2	43 (10.4)
3	206 (49.9)
Not Available	107 (25.9)
Total	425 (100)

**Table 4:** Summary of tumour size and age group comparison across the original patient cohort (*n* = 340). Includes cohort-wide descriptive statistics and comparisons between adult and paediatric groups. No significant difference was observed between groups.

Tumour Size Measurement	Value
**Overall**	
Tumour size (mean ± SD)	48.9 mm (±27.3 mm)
Tumour size (median, IQR)	44.0 mm (30–65 mm)
**By Age Group**	
Adult Mean	52.5 mm
Paediatric Mean	52.7 mm
Adult Median	46.0 mm
Paediatric Median	50.0 mm
Mann–Whitney U	W = 2966.5, *p* = 0.448

**Table 5:** Summary of symptom duration and age group comparison in the original patient cohort. Adults experienced significantly longer symptom durations before diagnosis than paediatric patients. Descriptive and comparative statistics are presented for the full cohort and by age group.

Symptom Duration Measure	Value
**Overall**	
Mean ± SD	11.4 ± 37.4 months
Median (IQR)	3 months (1–6 months)
By Age Group	
**Adult Mean**	13.2 months
Paediatric Mean	4.6 months
Adult Median (IQR)	4.0 (2.0–8.5)
Paediatric Median (IQR)	3.0 (1.3–4.5)
Mann–Whitney	W = 5178.5, *p* = 0.006

**Table 6:** Multivariable logistic regression results show odds of having a tumour >5 cm based on presenting symptoms in the original cohort. Statistically significant associations are bolded (*p* < 0.05). Odds ratios are shown with 95% confidence intervals.

Symptom	Odds Ratio (95% CI)	*p*-Value
Mass or Swelling	2.95 (1.78–5.02)	<0.001
Oral Symptoms	2.81 (1.24–6.50)	0.013
Pain	1.96 (1.15–3.35)	0.014
Nasal Symptoms	3.66 (1.24–11.0)	0.019
Aerodigestive tract obstruction	1.90 (0.93–3.86)	0.08
Trismus	2.58 (0.71–10.2)	0.15
Ulceration	1.49 (0.50–4.16)	0.46
Neurological Symptoms	1.34 (0.60–2.93)	0.47
Systemic Symptoms	1.54 (0.42–5.63)	0.51
Headaches	1.36 (0.35–4.82)	0.64
Orbital or Visual Symptoms	1.14 (0.46–2.71)	0.78

**Table 7:** Symptoms associated with tumour size (linear regression) in the original cohort. Coefficients from multivariable linear regression modelling tumour size (mm) as a function of presenting symptoms. Bolded values indicate *p* < 0.05.

Symptom	β (mm)	Std. Error	t-Value	*p*-Value
Mass or Swelling	8.33	4.97	1.68	0.096
Pain	5.02	4.56	1.10	0.272
Nasal Symptoms	14.58	9.30	1.57	0.119
Oral Symptoms	8.56	6.25	1.37	0.173
Trismus	11.26	10.53	1.07	0.286
Ulceration	−6.56	8.81	−0.75	0.457
Neurological	4.87	6.17	0.79	0.431
Systemic Symptoms	22.90	11.28	2.03	**0.044**

**Table 8:** Summary of linear and logistic regression analyses assessing whether log-transformed symptom duration predicts tumour size at diagnosis. Neither model demonstrated a statistically significant association.

Model Type	Outcome	Predictor	β/OR	*p*-Value	R^2^/AIC	Interpretation
Linear Regression	Tumour size (mm)	Log(Symptom Duration)	0.63	0.758	R^2^ = 0.001	Not significant
Logistic Regression	Tumour size > 5 cm	Log(Symptom Duration)	OR = 1.12	0.454	AIC = 274.4	Not significant

## Data Availability

Due to privacy and ethical restrictions, the raw data supporting this study cannot be made publicly available. Anonymised or aggregated data may be provided upon reasonable request to the corresponding author (samhita.shanmugasundaram.22@ucl.ac.uk).

## Abbreviations

The following abbreviations are used in this manuscript:

2D	Two-Dimensional
3D	Three-Dimensional
A&E	Accident and Emergency
AI	Artificial Intelligence
AIOM	Associazione Italiana di Oncologia Medica
BSG	British Sarcoma Group
BS	Bone Sarcoma
CBCT	Cone-Beam Computed Tomography
CI	Confidence Interval
COSS	Cooperative Osteosarcoma Study Group
CSCO	Chinese Society of Clinical Oncology
CT	Computed Tomography
DS	Dermal Sarcoma
EDI	Equality, Diversity and Inclusivity
EHR	Electronic Health Record
ESMO	European Society for Medical Oncology
FNCLCC	Fédération Nationale des Centres de Lutte Contre le Cancer
GOSH	Great Ormond Street Hospital
GP	General Practitioner
HN	Head and Neck
HNS	Head and Neck Sarcomas
IQR	Interquartile Range
JBI	Joanna Briggs Institute
KSSG	Korean Sarcoma Study Group
LSS	London Sarcoma Service
MDT	Multidisciplinary Team
MeSH	Medical Subject Headings
MPNST	Malignant Peripheral Nerve Sheath Tumour
MRI	Magnetic Resonance Imaging
NCCN	National Comprehensive Cancer Network
NGS	Next-Generation Sequencing
NHS	National Health Service
NICE	National Institute for Health and Care Excellence
NIHR	National Institute for Health and Care Research
NNT	Number Needed to Test
OM	Occipitomental
OPG	Orthopantomography
OR	Odds Ratio
PRISMA	Preferred Reporting Items for Systematic Reviews and Meta-Analyses
RIS	Radiation-induced Sarcoma
SBCO	Brazilian Society of Clinical Oncology
SD	Standard Deviation
STS	Soft Tissue Sarcoma
TNM	Tumour, Node, Metastasis
UCLH	University College London Hospitals
US	Ultrasound

## Appendix A

**Table A1:** Symptom counts and proportions in the original cohort.

Symptom	Count	Percentage (%)
Mass or Swelling	274	64.5
Pain	75	17.6
Ulceration	16	3.8
Oral Symptoms	27	6.4
Trismus	10	2.4
Aerodigestive Tract Obstruction	41	9.6
Nasal Symptoms	17	4.0
Orbital or Visual Symptoms	26	6.1
Systemic Symptoms	11	2.6
Asymptomatic	10	2.4
Not Described	53	12.5

**Table A2:** Median symptom duration by histological subtype.

Histological Subtype	n	Median (Months)	IQR
MPNST	11	7.0	3.0–84.0
Angiosarcoma	10	6.0	4.5–7.8
Chondrosarcoma	14	5.5	3.2–16.5
Spindle Cell Sarcoma	18	4.5	2.2–19.8
Ultra-Rare Sarcoma	20	4.0	2.0–7.0
Myxofibrosarcoma	6	3.5	2.2–5.5
Leiomyosarcoma	7	3.0	1.0–4.0
Liposarcoma	7	3.0	2.5–30.0
Osteosarcoma	55	3.0	2.0–7.0
Rhabdomyosarcoma	26	2.5	1.1–4.0
Radiation-Induced Sarcoma	20	2.0	1.0–4.2
